# Acute lung injury in mice associates with p44/42 and c-Jun N-terminal kinase activation and requires the function of TNFα receptor I

**DOI:** 10.1186/cc10701

**Published:** 2012-03-20

**Authors:** N Maniatis, A Sfika, I Nikitopoulou, A Vassiliou, C Magkou, M Kardara, A Armaganidis, C Roussos, G Kollias, S Orfanos, A Kotanidou

**Affiliations:** 1 University of Athens, Greece; 2 'Al. Fleming' Biomedical Sciences Research Center, Vari, Greece

## Introduction

Aspiration of hydrochloric acid-containing gastric juice leads to acute lung injury and hypoxemic respiratory failure due to an exuberant inflammatory response associated with pulmonary edema from increased endothelial and epithelial permeability. The aim of this study was to determine the role and signaling mechanisms of TNFα in experimental acute lung injury from hydrochloric acid aspiration using a combination of genetic animal models and pharmacologic inhibition strategies.

## Methods

Subjects were male and female C57Bl/6 mice, wild-type, TNFα knockout, TNFα receptor I knockout (*n *= 135). Hydrochloric acid was instilled intratracheally to mice, followed by respiratory system elastance measurement, bronchoalveolar lavage and lung tissue harvesting 24 hours post injection. The TNFα inhibitor etanercept was administered as pretreatment to a subset of mice prior to hydrochloric acid exposure.

## Results

Hydrochloric acid instillation induced an inflammatory response in the lungs of wild-type mice, evidenced as increased bronchoalveolar lavage total cells, neutrophils and total protein, histologic lung injury score and respiratory system elastance, while TNFα receptor I mRNA levels were maintained. These alterations could be prevented by pretreatment with etanercept or genetic deletion of the 55 kDa TNFα receptor I, but not by deletion of the TNFα gene. Hydrochloric acid induced a sixfold increase in apoptotic, caspase-3-positive cells in lung sections from wild-type mice, which was abrogated in mice lacking TNFα receptor I. In immunoblotting and immunohistochemistry studies, hydrochloric acid stimulated signaling via p44/42 and c-Jun N-Terminal kinase, which was blocked in TNFα receptor I knockout mice.

## Conclusion

Acute lung injury induced by intratracheal hydrochloric acid instillation requires the function of TNFα receptor I and associates with activation of downstream proinflammatory signaling pathways p44/42 and c-Jun N-Terminal kinase.

